# Atmosphere‐Snow Exchange Explains Surface Snow Isotope Variability

**DOI:** 10.1029/2022GL099529

**Published:** 2022-10-18

**Authors:** S. Wahl, H. C. Steen‐Larsen, A. G. Hughes, L. J. Dietrich, A. Zuhr, M. Behrens, A.‐K. Faber, M. Hörhold

**Affiliations:** ^1^ University of Bergen and Bjerknes Centre for Climate Research Bergen Norway; ^2^ Institute of Arctic and Alpine Research University of Colorado Boulder CO USA; ^3^ Alfred‐Wegener‐Institut Helmholtz Zentrum für Polar‐ und Meeresforschung Research Unit Potsdam Potsdam Germany; ^4^ University of Potsdam Institute of Geosciences Potsdam Germany; ^5^ Alfred‐Wegener‐Institut Helmholtz Zentrum für Polar‐ und Meeresforschung Research Unit Bremerhaven Bremerhaven Germany

**Keywords:** stable water isotopes, ice cores, snow‐atmosphere exchange, Greenland Ice Sheet, paleoclimate reconstruction, EGRIP

## Abstract

The climate signal imprinted in the snow isotopic composition allows to infer past climate variability from ice core stable water isotope records. The concurrent evolution of vapor and surface snow isotopic composition between precipitation events indicates that post‐depositional atmosphere‐snow humidity exchange influences the snow and hence the ice core isotope signal. To date, however, this is not accounted for in paeleoclimate reconstructions from isotope records. Here we show that vapor‐snow exchange explains 36% of the summertime day‐to‐day δ^18^O variability of the surface snow between precipitation events, and 53% of the δD variability. Through observations from the Greenland Ice Sheet and accompanying modeling we demonstrate that vapor‐snow exchange introduces a warm bias on the summertime snow isotope value relevant for ice core records. In case of long‐term variability in atmosphere‐snow exchange the relevance for the ice core signal is also variable and thus paleoclimate reconstructions from isotope records should be revisited.

## Key Points

- Atmosphere‐snow exchange of water vapor is a key process for the signal formation of the snow isotopic composition
- Taking into account isotopic fractionation during sublimation we explain a significant share of the observed day‐to‐day isotope variability
- Atmosphere‐snow exchange introduces a warm‐bias in the net summer snow isotope signal that is not accounted for in ice core interpretations

## 1 Introduction

Ice cores allow paleo‐climate reconstructions through climate proxies such as stable water isotope records. An accurate understanding of the transfer function between the climatic conditions and the ice core stable water isotope signal is thus crucial and important for integrating stable water isotope records in climate models. The rationale behind using ice core isotope records as climate proxies is the temperature dependency of the isotopic composition of precipitation that can be described by a Rayleigh distillation process. However, the assumption that the ice core climate signal is governed by the precipitation isotope signal alone has previously been challenged (Casado et al., 2021; Steen‐Larsen et al., 2014).

Besides uneven deposition through precipitation intermittency (Casado et al., 2020; Münch et al., 2021; Persson et al., 2011) and precipitation seasonality (Cuffey & Steig, 1998; Zheng et al., 2018), wind redistribution (Zuhr et al., 2021) and mixing with drifting snow (Stenni et al., 2016), post‐depositional processes are suggested to alter the isotopic composition of the snow. Specifically, vapor‐snow exchange processes occur continuously at the surface through turbulent humidity fluxes (Wahl et al., 2021) which are defined by local climate and below the surface during snow metamorphism. The latter is influenced by local climate through temperature shifts that induce temperature gradients in the snow and trigger interstitial vapor diffusion and associated snow recrystallization (Casado et al., 2021; Ebner et al., 2017; Touzeau et al., 2017). Thus, post‐depositional processes represent a potential mechanism behind the observed strong correlation between local temperature and the isotopic composition of snow and ice on various timescales other than Rayleigh distillation. In‐situ observations at ice core sites in both Greenland and Antarctica documented strong covariances of snow and vapor isotopic composition between precipitation events (Ritter et al., 2016; Steen‐Larsen et al., 2014). Furthermore, recent work demonstrated that isotopic fractionation during atmospheric vapor‐snow exchange drives the observed diurnal cycle in the near surface vapor isotopic composition (Madsen et al., 2019). Previously, such isotopic fractionation during sublimation, and hence influences of sublimation on the snow isotopic composition, have been disregarded as influential process due to low diffusivities of water molecules in the solid snow crystal matrix, which prevent mixing within the solid phase (Dansgaard, 1964; Friedman et al., 1991). However, laboratory and field experiments demonstrated isotopic enrichment of snow during sublimation (Hughes, Wahl, et al., 2021; Stichler et al., 2001), and in‐situ measurements of the sublimation flux's isotopic composition provided direct evidence for fractionation during sublimation (Wahl et al., 2021). The questions that remain include: (a) to which extent atmosphere‐snow isotope exchange processes are modifying the original precipitated snow isotope signal before the signal is buried and archived in ice records and, additionally, (b) which environmental factors are controlling the magnitude of the overprinting of the precipitation's original signal. In case of a significant fraction of the buried isotope signal stemming from such post‐depositional surface exchange processes, there is a need for revisiting existing paleoclimate reconstructions from ice core isotope records.

Here we address the role of atmospheric vapor‐snow exchange of water isotopes for the surface snow isotopic composition in polar areas. We make use of summertime observations obtained within the interior dry snow zone of the Greenland Ice Sheet (GrIS) that we perceive representative for other low accumulation sites. We focus on the effect of vapor‐snow exchange in the form of turbulent humidity fluxes during precipitation‐ and snowdrift‐free periods in summer and evaluate the impact on the summer seasonal isotope signal; the period in which we expect highest sublimation‐induced fractionation. To this end, a snow surface model is designed that is driven by in‐situ meteorological, snow and vapor observations with the objective to simulate the observed evolution of the snow surface isotopic composition and to identify the responsible drivers.

## 2 Materials and Methods

### 2.1 Field Site

Field observations were obtained at the East Greenland Ice Core Project (EastGRIP) field site on the GrIS (75°37′47′′N, 35°59′22′′W, 2700 m a.s.l in 2019). The estimated local accumulation rate is 10–15 cm water equivalent (w. eq.) year^−1^ (Karlsson et al., 2020). Measurements were taken in a dedicated clean‐snow area upwind from the main camp facilities. The data set presented here entails data of two summer campaigns, one between 11 May–5 August 2018, the other in the period 17 May–31 July 2019.

#### 2.1.1 Observations of Atmospheric Parameters

Continuous water vapor levels and corresponding stable water isotope ratios were measured simultaneously with a cavity ring‐down laser absorption spectrometer (CRDS) (Picarro L2140‐i). In 2018 (2019), the CRDS measured vapor at 1.8 m (2 m) above the snow surface for 15 min (30 min) every hour, and cycled through other measurement levels in the remaining time. Between 12:00 10 June and 21:00 1 July 2018 UTC the CRDS constantly measured at the 1.8 m level. Measurements were reported with ∼1 Hz frequency and given in volume mixing ratio (ppmv) for humidity and in δ‐notation (given ‰) for H_2_^18^O (δ^18^O) and ^1^H ^2^HO (δD) following Craig (1961):

(1) $$\delta^{*}=\frac{R^{*}}{R_{VSMOW}^{*}}-1$$

Where R* is the abundance ratio of rare (* = ^18^O or ^2^H) to abundant (^16^O or ^1^H) isotope and $R_{VSMOW}^{*}$ is the respective Vienna Standard Mean Ocean Water abundance ratio.

The CRDS measurements were calibrated for humidity dependency and referenced against the VSMOW‐SLAP scale (Text S1 in Supporting Information S1).

For the 2018 vapor data set 10 min periods were averaged yielding one measurement point per hour and a continuous 10 min timeseries between 10 June and 1 July. The 2019 data set contains 9 min averages amounting to three consecutive datapoints per hour. The resulting uncertainty on the averaged individual values is estimated to be 0.23‰ and 1.4‰ for δ^18^O and δD, respectively (Steen‐Larsen et al., 2013).

As a measure for vapor‐snow exchange, surface humidity fluxes were measured as latent heat fluxes (*LE*) using an eddy‐covariance (EC) system (Text S2 in Supporting Information S1) oriented against the prevailing wind direction of ∼240° (Wahl et al., 2021). The 1 standard deviation (SD) uncertainty on the *LE* measurement was conservatively estimated to be 20% (Litt et al., 2015; Vickers et al., 2010).

#### 2.1.2 Observations of Snow Parameters

For estimating the isotopic composition of the surface snow, consolidated daily snow samples were collected from eleven 10 m‐spaced and decorrelated (Stuart et al., 2021) locations along a 100 m long transect oriented in the prevailing wind direction. Per day, one sample was collected of the upper 0–0.5, 0–1, and 0–2 cm snow layer in 2018, and of the upper 0–0.5, 0–1, 0–2 and 0–5 cm in 2019 (Figure S1 in Supporting Information S1). The sample locations were marked to prevent collection of disturbed snow on the next day. The snow was sampled using an aluminum spatula, collecting equal amounts of snow at each location and transferring it to a plastic Whirl‐Pak® in which it was kept frozen throughout storage and transport. The top layer snow samples from 16 July 2018 and 23 July 2019 showed signs of evaporation during transport and were excluded from the analysis. The snow samples were melted in the closed Whirl‐Paks® to avoid evaporation and analyzed for their isotopic composition following van Geldern & Barth (2012) on a CRDS type Picarro L2140‐i at the stable water isotope laboratory of the Alfred‐Wegener‐Institute in Bremerhaven, Germany. The measurement accuracy on the individual sample of 2018 (2019) is 0.07‰ (0.06‰) and 0.11‰ (0.69‰) for δ^18^O and δD, respectively as estimated as average offset of a quality control standard. In addition to the daily snow samples, a high‐resolution (3 h) data set of snow samples (from three depths spanning 0–4 cm) for three periods of 2–3 days in 2019 is available (Hughes, Wahl, et al., 2021). Complementary to the isotope samples, snow density was measured at 10 locations along a different transect oriented perpendicular to the main wind direction by weighing snow samples of a known area and 2.5 cm thickness. Snow sampling and density measurements were performed roughly at the same time and thus share the same time information.

### 2.2 The Snow Surface Model

We use a numerical mass‐balance snow surface model (Figure S2 in Supporting Information S1) to simulate the evolution of the top (0–0.5 cm) snow layer's isotopic composition that we compare against observations. The model consists of a layered 5 cm snowpack (4 cm for the high‐resolution modeling). The vertical resolution is 0.5 cm with uniform snow density and temperature. At the time of a snow sampling event all model layers are initialized with the observed isotope values. For 2018, the deduced values of the 1–2 cm layers are used as initial conditions in the lowest 4 cm as, in contrast to 2019, no information is available of the snow isotopic composition below 2 cm. The snowpack's isotopic composition is then modulated by influences of surface fluxes affecting the top layer and isotope diffusion (Johnsen et al., 2000) affecting all layers. We use observations of meteorological and snow parameters as model forcing and reinitialized the model at the time of the next snow sampling event.

The time step of the model is 30 min, corresponding to the resolution of the *LE* observations. At every time step, an amount of snow *dm* (derived from *LE*) with a calculated isotopic composition (δ*_F_) is added (*LE* < 0) or removed (*LE* > 0) from the snowpack which modifies the isotopic composition of the top layer and the height of the snowpack.

During vapor deposition (*LE* < 0), δ_F_* is calculated from the 2 m vapor isotopic composition (δ_V_*) using the empirically derived linear equations of Wahl et al. (2021) to avoid assumptions of the effective supersaturation.

(2) $$\delta_{F}^{18_{O}}=1.50\cdot \delta_{F}^{18_{O}}+23.78‰$$

(3) $$\begin{array}{c} \delta_{F}^{D}=2.24\cdot \delta_{v}^{D}+445.20‰ \end{array}$$

During sublimation (*LE* > 0), δ_F_* is calculated depending on the experimental set up. Three different experiments are performed in which we vary the nature of the fractionation process during sublimation by implementing (i) kinetic fractionation with a Craig and Gordon (1965) (CG) approach, (ii) equilibrium fractionation $\left(R_{F}^{*}={\alpha_{eq}^{*}\left(T_{S}\right)}^{-1}\cdot R_{S}^{*}\right.$), or (iii) no fractionation (δ_F_ = δ_S_). For experiment (i) we parameterize the kinetic fractionation and calculate δ_F_* following Merlivat and Jouzel's (1979) formulation of CG:

(4) $$R_{F}^{*}=\frac{\left(1-k^{*}\right)}{\left(1-h\right)}\left(\frac{1}{\alpha_{eq}^{*}(T_{S})}R_{S}^{*}-hR_{V}^{*}\right)$$

(5) $$h=\frac{q_{2m}}{q_{sat}(T_{S})}$$

Where α_eq_ are ice‐vapor equilibrium fractionation coefficients from Majoube (1971) and Merlivat & Nief (1967), *k* ^18^ = 6‰ and *k* ^D^ = 0.88·*k* ^18^ are the kinetic fractionation factors, *R*_S_* is the isotopic composition of the top snow layer, *Ts* is the surface temperature and *h* is the humidity (*q*_*2m*_) deficit compared to the saturation humidity level at the surface (*q*_sat_). *Ts* and relative humidity (*h*) at each time step are calculated from *LE*, *q*_*2m*_ and the measured friction velocity following the bulk flux theory (Van AS, 2011). By making *h* and *T*_*s*_ dependent on *LE*, we reduce the uncertainty associated with observations of the surface parameters and ensure that the model input parameters satisfy basic physical principles.

The change in the top snow layer isotopic composition due to vapor‐exchange is calculated using a mass balance approach with a rate of change of

(6) $$\frac{\partial \delta_{S}^{*}}{\partial t}=\frac{s}{m}\left(\delta_{S}^{*}-\delta_{F}^{*}\right)$$

mwhere *s* is the sublimation rate calculated from *LE*, *t* is time and *m* is the mass of the top surface layer. For a full model description see Text S5 of the Supporting Information S1. We run the model for the periods 24 May–3 August 2018 and 23 May–29 July 2019 in which we have overlapping atmospheric and snow parameter observations. We compute the net change in δ‐values between daily sampling events (**Δ**δ*) and compare the model results ($\Delta \delta_{\mathrm{model}}^{*}$) against the observed changes ($\Delta \delta_{\mathrm{obs}}^{*}$). We compute the ratio of explained variance (*VE*) from the 1:1 line as:

(7) $$\mathrm{VE}=1-\frac{\sum {\left(\Delta \delta_{\mathrm{obs}}^{*}-\Delta \delta_{\mathrm{model}}^{*}\right)}^{2}}{\sum {\left(\Delta \delta_{\mathrm{obs}}^{*}-\bar{\Delta \delta_{\mathrm{obs}}^{*}}\right)}^{2}}$$

Of the 158 (84 in 2018/74 in 2019) consolidated snow surface samples collected, 48 (30/18) samples were influenced by snowfall and are therefore excluded from the analyses. Likewise, 20 modeling periods in which max wind speeds of >10 ms^−1^ were observed were excluded to prevent influences from drifting snow. In the remaining model periods, the maximum 30 min averaged wind speed did not exceed 5.5 ms^−1^, which is in compliance with literature thresholds for blowing snow (Birnbaum et al., 2010).

#### 2.2.1 Sensitivity Analysis

We identify the most influential environmental drivers for the model results by performing a sensitivity analysis. For this we varied the input parameters δ_V_, *LE* and the initial snow isotopic composition by ±1 SD and ±2 SD, where SD is the uncertainty estimate (Table S2 in Supporting Information S1), and evaluated changes in RMSE and *VE*. Additionally, the kinetic fractionation coefficient *k* was varied and the uncertainty in the sampling time accounted for by adding ±2 h (in 30 min steps). We then calculate the SD in the set of resulting **Δ**δ* for each tested parameter and calculate the median of all modeling periods. We rank the model's sensitivity to the input parameters by the resulting SD of the medians. The results are given in Table S2 and Figure S4 in Supporting Information S1.

#### 2.2.2 Uncertainty Estimates of Model Result

For estimating the uncertainty of the modeled **Δ**δ*, we performed 1,000 Monte Carlo simulations for each day‐to‐day modeling period by adding random noise to the input parameters *LE*, δ_V_, initial δ_S_ and atmospheric humidity *q*. The resulting distribution's SD is taken as modeling uncertainty and visualized as error bars in Figure 3. Text S3 in the Supporting Information S1 explains the estimated uncertainty level for each model parameter.

## 3 Results

### 3.1 Vapor and Snow Interplay

**Figure 1:**
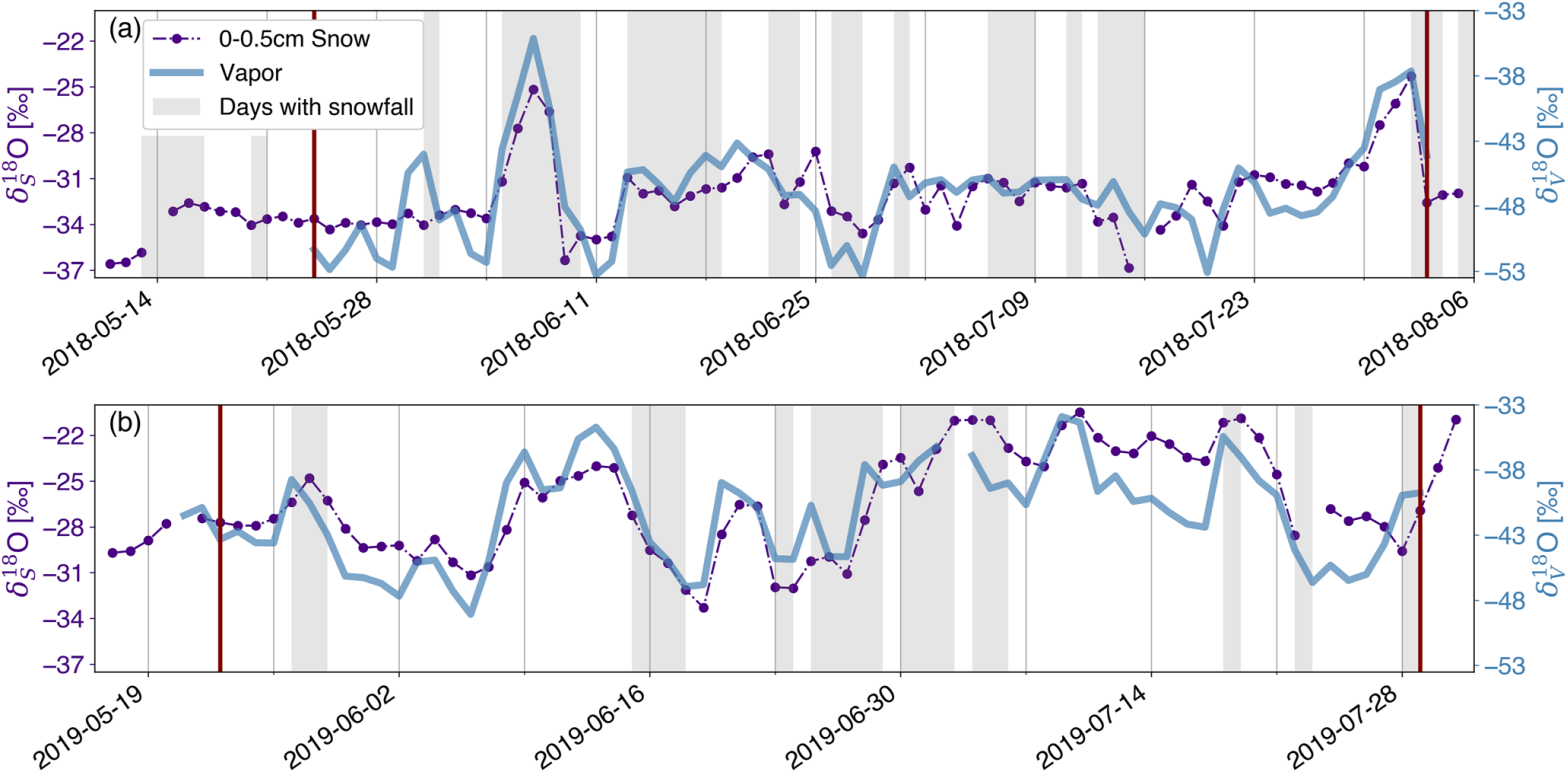
Observations of seasonal snow surface and vapor isotope variability. Shown are timeseries of the δ_S_^18^O signal of daily consolidated samples (purple circles) and the daily 2 m vapor isotope signal δ_V_^18^O (solid blue line) in the two observation periods in 2018 (a) and 2019 (b). Days with recorded snowfall are grayed out. The red vertical lines frame the modeling periods.

The daily consolidated surface (0–0.5 cm) snow samples show covarying temporal evolution with daily mean 2 m vapor isotopic composition (Figure 1). The snow isotopic composition (δ_S_) of the two observation periods was distinct, with more enriched values in 2019 compared to 2018 (Table S1 in Supporting Information S1). Similarly, the 2 m water vapor (δ_V_) was more enriched in 2019 compared to the 2018 summer season. The snow surface isotope signal was more variable over time with a higher standard deviation in 2019 compared to 2018.

Substantial changes in the isotopic composition of the snow corresponding to synoptic weather regime changes were recorded not only during, but also between precipitation events. Both isotopic enrichment and depletion events spanning several days were observed.

Throughout both summer observation periods, the recorded humidity flux amounted to a net sublimation with ∼3 mm (5 mm sublimation and 2 mm deposition) w. eq. in 2018 and ∼5 mm (7 mm sublimation and 2 mm deposition) w. eq. in 2019.

### 3.2 Snow Model Performance on Sub‐Daily Timescales

To quantify the role of atmospheric vapor‐snow exchange for the snow isotope signal, we simulate the evolution of the snow surface isotopic composition at the field site. First, we use the high‐resolution snow data set (observations of snow surface isotopic composition every 3 hours) to investigate the model performance on sub‐daily timescales and thus to evaluate sublimation and deposition periods separately.

**Figure 2:**
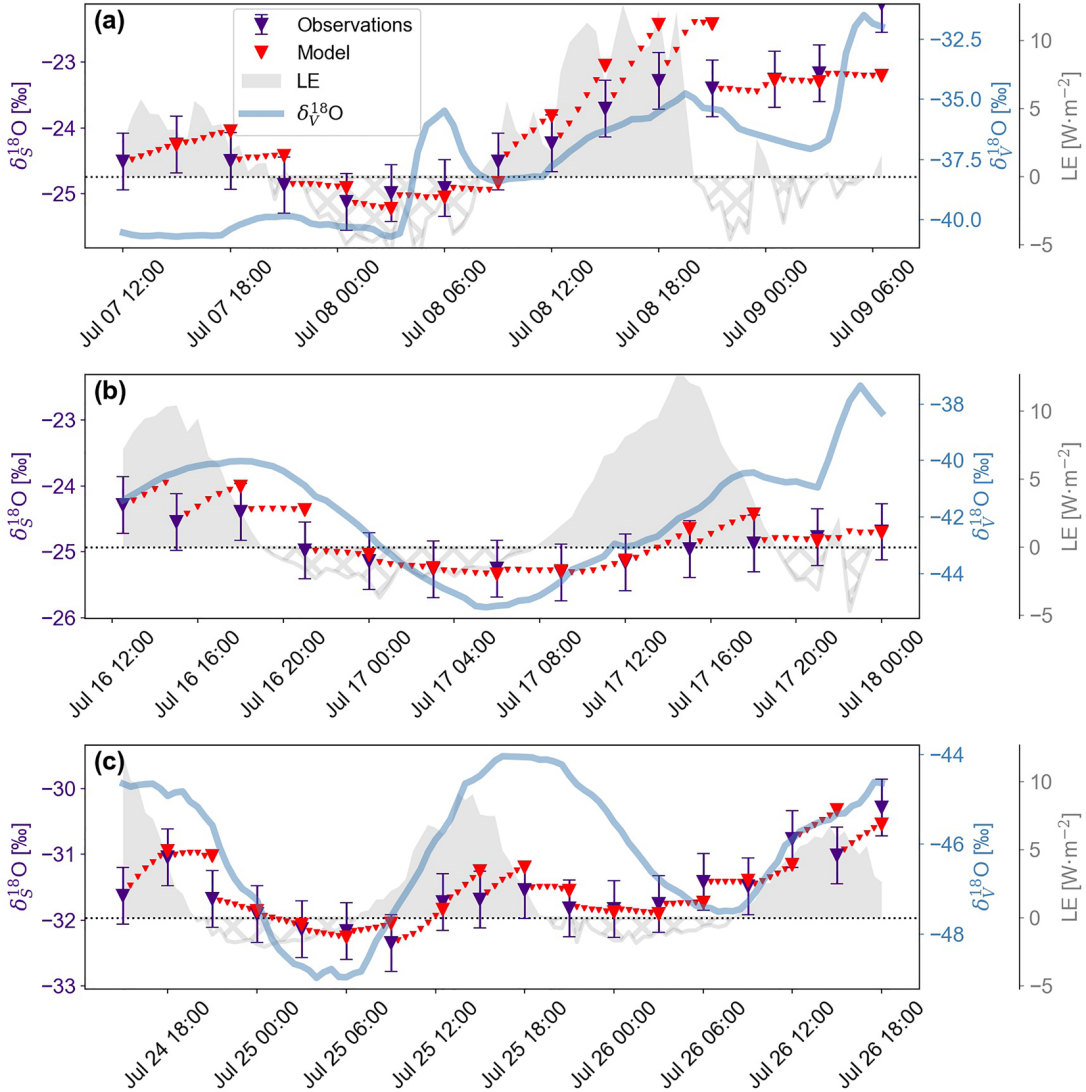
Simulations and observations of sub‐daily snow surface isotope variability. The simulated evolution (small red triangles) and the compared values (bigger red triangles) of the δ_S_ signal during the high‐resolution snow sampling periods are shown. The observations (purple triangles, error bars show SD between samples from three locations) serve as model validation. The model is reinitialized at every observation point and run until the next available observation point. The forcing parameter *LE* with deposition (hatched) and sublimation (shaded) phases is shown in gray. The 2 m vapor isotopic composition (δ_v_) is shown as blue line. Pearson correlation coefficients for a, b, and c are *r* = 0.83, *r* = 0.86, *r* = 0.83, respectively. Time is UTC.

Figure 2 shows that the model is able to capture the observed snow evolution on sub‐daily timescales qualitatively during both snow isotopic enrichment and depletion. The pearson correlation coefficients (*r*) between modeled and observed values are *r* ≥ 0.83 for all three periods. However, isotopic enrichment during sublimation (*LE* > 0) is slightly overestimated compared to observations. Despite the simple empirical linear relationship used for the deposition conditions, the modeled isotopic depletion during deposition periods (*LE* < 0) is consistent with observations.

### 3.3 Modeling of Snow Surface Day‐to‐Day Isotope Variability

**Figure 3:**
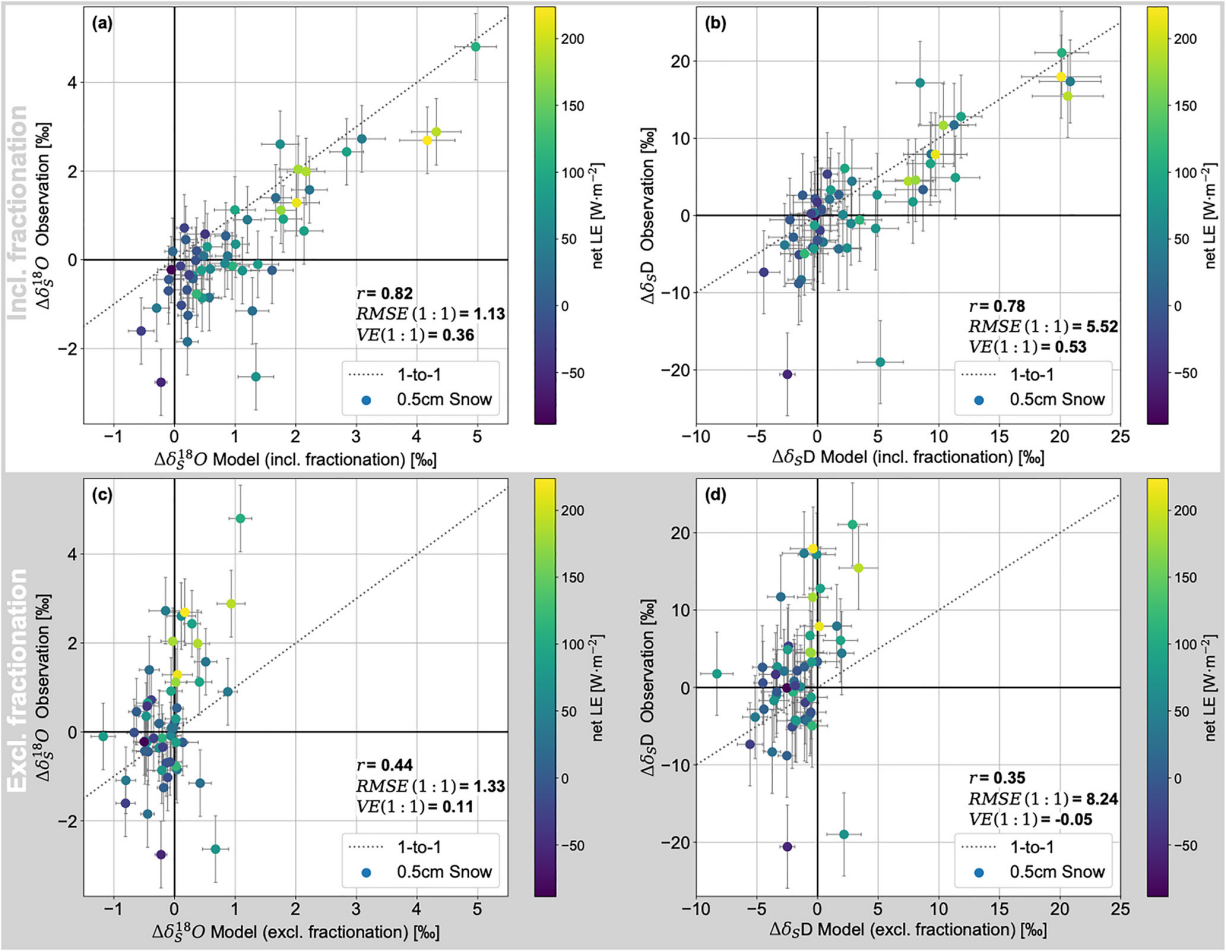
Modeled versus observed day‐to‐day change in snow isotopic composition. The modeled day‐to‐day change in snow isotopic composition (δ^18^O in (a) and δD in (b)) using kinetic fractionation (experiment i) is plotted against the observed day‐to‐day change. The simulation with no fractionation during sublimation (experiment iii) is shown for δ^18^O and δD in (c) and (d), respectively. All model periods (*N* = 53) are color coded according to the cumulative *LE* observed between the respective two sampling events, bracketing the model period. 1‐to‐1 lines are shown in all plots. The uncertainties on the observations are inferred as square‐summed standard error of the mean whereas the uncertainties on the model results were estimated using a Monte Carlo approach.

Next, we make use of the full span of simultaneous vapor and snow isotope measurements of the two field campaigns and simulate the change of the snow isotopic composition in between daily snow sampling events, which will be referred to as day‐to‐day variability. We compare our modeled day‐to‐day variability of snow isotopic composition to the in‐situ observed changes. Results of the experiment including kinetic fractionation (i) are shown in Figure 3 for **Δ**δ^18^O and **Δ**δD in (a) and (b), respectively. A one‐to‐one line is shown to indicate model performance. Based on the *VE* calculation (Equation 7), the model explains 36% of the variance in δ^18^O observed in the top snow layer on days without snowfall and wind drift and 53% of the δD variance. Both model and observations show that higher changes in snow surface isotopic composition coincide with higher net sublimation.

When excluding both equilibrium and kinetic fractionation during sublimation and allowing for diffusion and deposition processes only (iii), the *VE* values for both isotopic species approach zero (Figures 3c and 3d). If equilibrium fractionation (ii) is used instead of a CG type model the *VE* values are 0.30 for δ^18^O, and 0.25 for δD, respectively (Figure S3 in Supporting Information S1). See Table S2 in Supporting Information S1 for an overview of the performance metrics for all experiments and an experiment without isotope diffusion.

#### 3.3.1 Uncertainty Evaluation

The model result (**Δ**δ_S_) is most sensitive to uncertainties in the initial snow isotopic composition, followed by *LE*, δ_V_ and the timing of snow sampling (Figure S4 in Supporting Information S1). Model sensitivity for normalized relative humidity (*h*) and surface temperature (*Ts*) were not tested individually, since they are calculated from *LE*. Generally, variability in the perturbation response was higher for periods with higher observed **Δ**δ_S_ values. Hence the relative importance of vapor‐snow exchange processes on the snow isotopic composition varies throughout the season. Figure 2 shows that the surface snow model slightly overestimates the enrichment due to sublimation. The overestimation is counteracted by reduced *LE* or shortened modeling periods and explains the increased *VE* in the respective sensitivity model runs (Table S2 in Supporting Information S1). Similarly, the model performs better when vapor or snow isotopic composition are perturbed toward equilibrium conditions, that is, through vapor depletion or snow enrichment. Approaching equilibrium, the gradient between snow and vapor isotopic composition is reduced, which reduces the effect of kinetic fractionation in the snow.

## 4 Discussion

### 4.1 Evaluation of the Model Performance

To draw conclusions about the dominant processes at play, we compare the experiments in terms of *VE* values and spread of data points around the one‐to‐one lines. We find that experiment (i), including kinetic fractionation during sublimation, performed best albeit overestimating the isotopic enrichment. Experiment (ii) with equilibrium fractionation performed moderately but underestimated the enrichment, wheras experiment (iii) without fractionation during sublimation performed worst. The improved model performance for δD compared to δ^18^O in (i) can be explained by the increased sensitivity of δ^18^O to kinetic effects (Table S2 in Supporting Information S1). The model results do not change significantly when isotope diffusion is disabled (Table S2 in Supporting Information S1) which demonstrates that the key process responsible for the model performance is the magnitude of isotopic fractionation during sublimation. However, the overestimation in experiment (i) suggests that the CG approach might be sub‐optimal when simulating the fractionation process during snow sublimation. One way to improve model fidelity might be the introduction of a temperature‐dependency which impacts the magnitude of fractionation. It has been suggested that temperature controls whether the diffusion within the solid snow matrix is effective enough to allow homogenization of the remaining snow crystal and thus controls the imprint of the fractionation signal (Hu et al., 2022). In other words, temperature can control snow metamorphism and thus impacts the fractionation strength. Additionally, ambient temperature shifts can establish snow temperature gradients and thus induce sub‐surface vapor fluxes from below the 5 cm model domain (Casado et al., 2021) that can affect the surface layer. Whether it is reasonable to include a temperature dependency when simulating sublimation‐fractionation will be subject of future studies.

### 4.2 Climate Signal Formation Through Vapor‐Snow Exchange

Our results show that by incorporating kinetic fractionation during sublimation, the snow surface model is able to explain a substantial amount of the to‐date unexplained observed variability in the surface snow isotope signal. By adapting the CG type sublimation model, we allow for feedbacks from the vapor isotopic composition on the magnitude of sublimation effects (Gat, 1996). Since the vapor isotopic composition is modulated by temperature‐dependent Rayleigh distillation and synoptic weather events (Bagheri Dastgerdi et al., 2021; Steen‐Larsen et al., 2013) it is a climate proxy itself. Vapor‐snow exchange constitutes a pathway for how the atmospheric climate signal in the vapor isotopic composition can be imprinted on the snow isotope signal during precipitation‐free periods. During sublimation it is the gradient between vapor and snow isotopic composition that, together with the sublimation rate, defines the magnitude of sublimation effects in the snow, which is visible in the sensitivity analysis of this study. Thus, we argue it is this isotopic gradient, that is, the disequilibrium between the actual vapor signal and vapor in equilibrium with the snow, that is the main driver for the flux isotopic composition (Benson et al., 1994), and is consequently important for the magnitude and direction of changes in the snow isotope signal. During deposition the vapor isotopic composition directly defines the deposition flux isotope signal and thus affects the snow signal (Casado et al., 2016; Wahl et al., 2021).

### 4.3 The Effect of Sublimation for the Summer Isotope Signal

For evaluating the importance of sublimation for the summer signal in the snowpack isotope record the comparison of the precipitation versus the sublimation signal both in terms of mass balance and preserved signal seems necessary. However, with no information about the precipitation's isotopic composition and mass (Zuhr et al., 2021), this comparison is infeasible. Instead, we calculate the observed change in snow isotopic composition during the observation periods and exclude days with snowfall or wind redistribution. The observations show a net enrichment in snow isotopic composition during fresh snow‐free periods in both 2018 and 2019 (Table S1 in Supporting Information S1). Performing the same calculation on the no fractionation simulation (iii) data set yields a net depletion over the observation period due to deposition and diffusion influences (Table S1 in Supporting Information S1). The offsets between both calculations of ∼14‰ and ∼6‰ for δ^18^O in 2018 and 2019, respectively demonstrate that sublimation significantly enriches the snow that is in contact with the atmosphere. Note that the calculated values can not be interpreted as the winter‐summer amplitude in the δ^18^O signal which is observed in the range of 10–15‰ (Komuro et al., 2021). Instead, it is the sublimation contribution to the summer signal, which is overlaid by variability emanating from precipitation. However, it demonstrates that vapor‐snow exchange at EastGRIP introduces a warm‐bias in the summer isotope signal through sublimation and therefore affects the annual isotope value.

### 4.4 Significance of Vapor‐Snow Exchange on Longer Timescales

Whether vapor‐exchange processes imprint on the snowpack on seasonal to annual timescales thus depends on the local reoccurrence of precipitation events, that is, the length of precipitation free periods, the magnitude and net direction of the surface humidity flux and the disequilibrium between vapor and snow surface. Since those climate characteristics are likely variable both spatially and temporally, the importance of vapor‐snow exchange on the snow isotopic composition is not constant. Our findings suggest that variability in ice core isotope records could include a vapor‐snow exchange component rather than a precipitation signal alone. This is particularly relevant for seasonal‐amplitude climate studies but also for studies of longer time scales when the overall magnitude of the humidity flux changes, for example, during glacial and interglacial periods. Hence paleoclimate interpretations of ice core isotope records (e.g., Kawamura et al., 2017; Masson‐Delmotte et al., 2005; Steffensen et al., 2008) should be revisited in the context of a warm‐bias introducing vapor‐snow exchange.

## 5 Conclusions

We investigated the summertime snow isotope variability on the GrIS that is the foundation for ice core isotope records which are interpreted as climate proxies. We set up a simple snow model of the upper 5 cm of the snow column and forced it with observations of surface fluxes, snow and the isotope signal of atmospheric water vapor. By modeling the evolution of the snow surface isotope signal we investigated the exchange of water isotopes between the snow and atmosphere as a snow isotope signal formation process. Our results showed that by incorporating kinetic fractionation during sublimation we can explain a substantial amount of the day‐to‐day variability that is observed in the surface snow isotopic composition in between precipitation events. In contrast, when omitting isotopic fractionation during sublimation the model fidelity decreased drastically which supports existing studies of a fractionation‐inducing sublimation process. A comparison between modeled and observed net change of the snow isotope signal over the season demonstrated that atmospheric vapor‐snow exchange has an enriching effect on the summer snow that can be attributed to sublimation, that is, sublimation can introduce a warm‐bias in climate reconstruction studies when ice core water isotope records are interpreted without accounting for vapor‐snow exchange. Considering that atmosphere‐snow exchange processes are not constant in time, the impact on the snow isotope signal has likely varied in the past. Thus we suggest to revisit climate reconstruction studies from ice core stable water isotope records in the framework of a summer snow isotope signal that is partly defined by atmosphere‐snow exchange.

## Supporting information

Supporting Information S1

## Data Availability

The observational isotopic and meteorological data sets used as input for the numerical model and as validation reference in the study are available in the PANGAEA data repository under the CC‐BY licence. Data sets of the water vapor isotope measurements and surface fluxes are available as Steen‐Larsen et al. (2022) and Steen‐Larsen and Wahl (2021a, 2021b, 2022a). The snow isotope data is available as Hörhold et al. (2022a, 2022b) and Hughes, Steen‐Larsen, et al. (2021). The snow density and snow temperature datasets are available as Steen‐Larsen et al. (2022a) and Steen‐Larsen and Wahl (2022b). A basic version of the snow surface model is published on Zenodo at https://doi.org/10.5281/zenodo.7117642.
