# Supplementary material for: Atmosphere‐Snow Exchange Explains Surface Snow Isotope Variability
Source: Geophys Res Lett. 2022 Oct 18;49(20):e2022GL099529. doi: 10.1029/2022GL099529 (PMC9787379; doi:10.1029/2022GL099529)
Supplement: Supplementary file 1 — Supporting Information S1 [file GRL-49-e2022GL099529-s001.pdf]

AGU PUBLICATIONS

Geophysical Research Letters

Supporting Information for

Atmosphere-Snow Exchange Explains Surface Snow Isotope Variability

S. Wahl[1], H. C. Steen-Larsen[1], A. G. Hughes[2], L. J. Dietrich[1], A. Zuhr[3], M. Behrens[4], A.-K. Faber[1], M. Hörhold[4]

[1] University of Bergen and Bjerknes Centre for Climate Research, Bergen, Norway.

[2] Institute of Arctic and Alpine Research, University of Colorado, Boulder, CO 80309-0450 USA.

[3] Alfred-Wegener-Institut Helmholtz Zentrum für Polar- und Meeresforschung, Research Unit Potsdam, Potsdam, Germany and University of Potsdam, Institute of Geosciences, Potsdam, Germany.

[4] Alfred-Wegener-Institut Helmholtz Zentrum für Polar- und Meeresforschung, Research Unit Bremerhaven, Bremerhaven, Germany.

Corresponding author: Sonja.wahl@uib.no

Contents of this file

Text 1 - Text 5
Figures S1 to S5
Table S1 – S2

Introduction

This Supplementary Material includes some detailed complementary information to the main text that may be of interest to some readers. The two field campaigns are compared in terms of vapor and snow isotopic composition in Table S1 and Figure S1. The calibration protocol for the CRDS water vapor isotope measurements is given in Text 1. Details on the Eddy-Covariance systems are given in Text 2. The noise level estimates used in the Monte Carlo simulations for the model uncertainty estimate are outlined in Text 3. Text 4 and Figure S5 explain how we estimated the uncertainty of the snow surface isotopic composition. The snow surface model is described in more detail in Text 5 and Figure S2 while the results of the model sensitivity study are shown in Figure S4 and detailed values are given in Table S2. Finally, the results of the experiment using equilibrium fractionation (ii) during the sublimation process are shown in Figure S3.

Text 1: Calibration protocol for CRDS measurements of water vapor

The CRDS measurements were corrected for the instrument's humidity dependency and calibrated against the VSMOW-SLAP scale following the approach of Steen-Larsen et al. (2013). Calibrations were performed every day using vaporized secondary standards at a constant humidity level. Secondary standards were provided by the Institute of Arctic and Alpine Research, University of Colorado and bracketed the observed range of δ-values. Since the CRDS was observed to be stable within the individual field campaigns, all calibration runs were grouped together to obtain one set of VSMOW-SLAP calibration curves for δD and $\delta^{18}O$ per campaign. The CRDS' humidity dependency was corrected using humidity calibrations carried out several times throughout the campaigns. The humidity dependency curves were established for each standard by providing constant vapor streams at different humidity levels. The humidity dependency response curves for each campaign were linearly interpolated in the δ-space to account for the dependency of the response curves on the standard's isotopic composition. The humidity mixing ratio of the CRDS was calibrated against a custom-made dew-point generator for a temperature range between –40°C and 0°C in the laboratory.

Text 2: Specifications of the eddy-covariance systems

In 2018, the system consisted of a CSAT3 sonic anemometer and a Krypton Hygrometer (KH20) both from Campbell Scientific (CS). Latent heat flux (LE) estimates for 2018 were calculated for averages of 10min using a custom processing software and post-processed to 30min means. In 2019, 30min resolution surface fluxes were measured using a CS Irgason system and the processing software EasyFlux which follows established correction protocols (e.g. Mauder et al., 2006).

Text 3: Uncertainty estimate of modeled Δδ using Monte Carlo simulations

For estimating the uncertainty of the modeled **Δ**δ*, we performed 1000 Monte Carlo simulations for each day-to-day modeling period by adding random noise to the input parameters *LE*, δ_V, initial δ_S and atmospheric humidity *q*.

For *LE*, we account for EC measurement accuracy errors by multiplying the input timeseries with a factor drawn from a normal distribution around a mean of 1 and SD 20%. The parameters *h* and *Ts* are calculated from *LE* subsequently. For the other input parameters, we account for the uncertainty of the measurements by adding noise to the individual datapoints drawn from a normal distribution with mean 0 and a representative SD for the respective parameter, i.e. SD of δ_v is 0.23‰ for $\delta^{18}O$ and 1.4‰ for δD (Steen-Larsen et al., 2013). Due to the absence of a manufacturer precision statement for the CRDS humidity readings, we infer a relative error of 0.2% based on the calibration pulses. Finally, we define the uncertainty of the estimated mean snow surface isotopic composition δ_S from an additional snow sample set as 0.53‰ and 3.8‰ for $\delta^{18}O$ and δD, respectively (Text 1). Since **Δ**δ* is the subtraction of two consolidated snow samples we propagate the error to find the uncertainty on the observed **Δ**δ* to be 0.74‰ for $\delta^{18}O$ and 5.4‰ for δD which are indicated as error bars in Figure 3 in the main text.

Text 4: Estimating the uncertainty of the average 0.5cm snow-layer isotopic composition

To estimate the uncertainty associated with the consolidated snow surface isotopic composition sample as representative value for the mean snow surface isotopic composition we make use of snow samples collected from ten positions along a 90m long transect perpendicular to the prevailing wind direction. Snow of the upper 0-0.5cm layer was sampled with a 10m distance between sampling locations. The sampling scheme was repeated on 30 days in 2019. We calculate the deviation of each individual sample from the respective sample mean and calculate the standard deviation of all data points. The distribution is shown as histogram in Fig. S5.

This yields an uncertainty on the consolidated snow isotopic composition of e=0.53‰ for $\delta^{18}O$ and e=3.8‰ for δD.

Text 5: Snow surface model

The evolution of the snow surface isotopic composition is simulated by setting up a snow surface model using a mass balance approach. At initializing, a $H=5$ cm thick snowpack domain is divided into ten layers with $j = 1$ being the surface layer in contact with the atmosphere. The mass (m) per unit surface area of the surface layer is calculated using the daily observed snow densities (ρ_S).

Vapor-Snow Exchange

At every time step (i), with $dt=30$ min resolution, an amount of mass (dm) is either removed or added to the snowpack during sublimation ($LE > 0$) or deposition ($LE < 0$) conditions, respectively. The LE measurements together with the latent heat of sublimation ($L_s = 2.834e^6$ J kg^{-1}) are used to calculate the sublimation rate (s in kg m^{-2} s^{-1}), from which dm is calculated following:

$$s = \frac{LE}{Ls} \cdot \rho_S \qquad (1)$$

$$dm = s \cdot dt \qquad (2)$$

The mass balance per time step equals:

$$R_s^{i+1} = \frac{m^i R_S^i - dm R_F^i}{m^i - dm} \qquad (3)$$

With R_S and R_F being isotopic ratio of snow and humidity flux, respectively.

$$R_S^{i+1} = \frac{m^i R_S^i}{m^{i+1}} - \frac{dm R_F^i}{m^{i+1}} \qquad (4)$$

$$R_S^{i+1} - R_S^i \quad = R_S^i\left(\frac{m^i}{m^{i+1}}\right) - R_S^i - R_F^i\left(\frac{s}{m^{i+1}}\right)dt \tag{5}$$

$$\frac{R_S^{i+1} - R_S^i}{dt} \quad = \frac{R_S^i\left(\frac{m^i}{m^{i+1}} - 1\right)}{dt} - R_F^i\left(\frac{s}{m^{i+1}}\right) \tag{6}$$

Substituting $m^{1+i} = m^i - dm = m^i - (s \cdot dt)$ yields:

$$\frac{R_S^{i+1} - R_S^i}{dt} = R_S^i\left(\frac{s}{m^{i+1}}\right) - R_F^i\left(\frac{s}{m^{i+1}}\right) = \frac{s}{m^{i+1}}(R_S^i - R_F^i) \tag{7}$$

From this we can find the partial time (t) derivative for small mass changes:

$$\frac{\partial R_S}{\partial t} = \frac{s}{m}(R_S - R_F) \tag{8}$$

Converted to δ-notation in ‰ this equals:

$$\frac{\partial \delta_S}{\partial t} = \frac{s}{m}(\delta_S - \delta_F) \tag{9}$$

The isotopic composition of the sublimation flux is computed in analogy to the evaporation flux as formulated by Merlivat and Jouzel (1979):

$$R_F \quad = \frac{(1-k)}{(1-h)}\left(\frac{1}{\alpha_{eq}(T_S)}R_S - hR_v\right) \tag{10}$$

$$h \quad = \frac{q_v}{q_{sat}(T_S)} \tag{11}$$

Where h is the saturation deficit, i.e. $(1-h)$ is the humidity (q) gradient inducing the humidity flux. δ_v and q_v are the isotopic composition of the vapor and the humidity at the 2 m level. The surface is assumed to be at saturation $q_{sat}(T_S)$ with regards to the surface temperature T_S. T_S, and hence q_{sat} and h, are calculated from LE and q_v solving a humidity flux bulk equation following Van As (2011) with stability correction functions for stable (Holtslag & De Bruin, 1988) and for unstable (Paulson, 1970) conditions. We use the median of observed roughness length (z_0) values during neutral atmospheric stability in 2019 as constant momentum roughness length of $z_0=1.3\cdot10^{-4}$m and calculate the scalar roughness length from it following Andreas (1987). $\alpha_{eq}(Ts)$ and k are the equilibrium and kinetic fractionation coefficients, respectively with $k^{18O} = 6$ ‰ and $k^D = 0.88 \cdot k^{18O}$. Ice-vapor equilibrium fractionation factors of Majoube (1971) and Merlivat & Nief (1967) are

used for the sublimation flux and any other occasion of equilibrium fractionation. During times of $0.98 < h < 1$ the snow isotopic composition is not changed by the sublimation flux since δ_F approaches unrealistic values.
During deposition conditions, we use the empirically derived linear functions of Wahl et al. (2021) to calculate the humidity flux isotopic composition from the 2m vapor isotopic composition.:

$$\delta_F^{18O} = 1.50 \cdot \delta_V^{18O} + 23.78‰ \tag{12}$$

$$\delta_F^{D} = 2.24 \cdot \delta_V^{D} + 445.20‰ \tag{13}$$

The vapor-snow humidity exchange only affects the isotopic composition of the surface layer and its mass. Additionally, a diffusion model is realized, that modulates the isotopic composition along the gradients within the air space of all layers within the 5 cm snowpack at every time step.

Isotope Diffusion in Snow

Following Johnsen et al. (2000), the diffusivity (Ω) of water vapor in air (a) in $cm^2 s^{-1}$ is given by reference ($P_0 = 1\,atm$) and ambient pressure (P) and absolute ($T_0 = 273.15$ K) and snow temperature (T). T of the entire 5cm snowpack model domain is calculated for every time step as average from in-situ observed snow temperatures at 10cm depth and calculated Ts.

$$\Omega_a = 0.211 \left(\frac{T}{T_0}\right)^{1.94} \left(\frac{P_0}{P}\right) \tag{14}$$

Ratios of isotope diffusivities are taken from Merlivat (1978) which yields diffusivities of the isotope species (Ω_{a*}) in air with:

$$\Omega_{a^{18}O} = \frac{\Omega_a}{1.0251}$$

$$\Omega_{aD} = \frac{\Omega_a}{1.0285}$$

In snow, the pore space shape limits the diffusion, which can be described with the tortuosity factor (τ), that is assumed to be a function of density (Schwander et al., 1988):

$$\frac{1}{\tau} = 1 - 1.3 \cdot \left(\frac{\rho_S}{\rho_{ice}}\right)^2 \tag{15}$$

with density of ice $\rho_i = 917\,kg\,m^{-3}$. The diffusivity in firn (here snow) can then be calculated with molar mass of water (mo), saturation vapor pressure over ice (p) from Goff & Gratch (1946) and the gas constant $R = 8.314\,Jmol^{-1}K^{-1}$:

$$\Omega_{f^*} = \frac{mo\,p\Omega_{a^*}}{RT\alpha^*\tau}\left(\frac{1}{\rho_S} - \frac{1}{\rho_{ice}}\right) \tag{16}$$

Diffusion of isotopes within the snow pack can therefore be described by:

$$\frac{\partial \delta^*}{\partial t} = \Omega_{f^*} \frac{\partial^2 \delta^*}{\partial z^2} \tag{17}$$

Variable Snow Height

To account for mass transfer and resulting changes in snow pack height, we introduce the dimensionless height (ξ), defined by the depth profile (z) and H:

$$\xi \; = \frac{z}{H(t)} \tag{18}$$

$$\partial z = H \cdot \partial \xi \tag{19}$$

Equation (17) then reads:

$$\frac{\partial \delta^*}{\partial t} = \frac{\Omega_{f^*}}{H^2} \left(\frac{\partial^2 \delta^*}{\partial \xi^2} \right) \tag{20}$$

This way the mass gain or loss are equally distributed over all snowpack layers which preserves a surface layer close to the original 0.5cm and thus prevents an artificial enhancement of the sublimation effect in case of high net sublimation, i.e. high mass removal which would otherwise only thin the top layer.

Full Snow Surface Model

The Forward Euler scheme is used to model the evolution of the snow isotopic composition in all snow layers resulting from influences of diffusion (equation 20) and surface fluxes (equation 9, only affecting surface layer). The full model equation with matrix A for diffusion impact on all layers

$$A = \begin{bmatrix} -1 & 1 & 0 & \dots & \dots & \dots & 0 \\ 1 & -2 & 1 & \ddots & & & \vdots \\ 0 & 1 & -2 & 1 & \ddots & & \vdots \\ \vdots & \ddots & 1 & -2 & 1 & \ddots & \vdots \\ \vdots & & \ddots & \ddots & \ddots & \ddots & \vdots \\ \vdots & & & \ddots & \ddots & \ddots & \ddots \\ 0 & \dots & \dots & \dots & \dots & 1 & -1 \end{bmatrix}$$

and B for surface flux influences on top layer j=1:

$$B = \begin{bmatrix} 1 \\ 0 \\ \vdots \\ 0 \end{bmatrix}$$

then reads for either isotope species δ^* and timestep *i*:

$$\delta^{i+1} = \delta^i + \frac{\Omega_{fi}}{H^2} \frac{1}{\Delta \xi^2} \delta^i [A] dt + \frac{sr}{m} (\delta_{j=1} - \delta_F)[B] dt \tag{23}$$

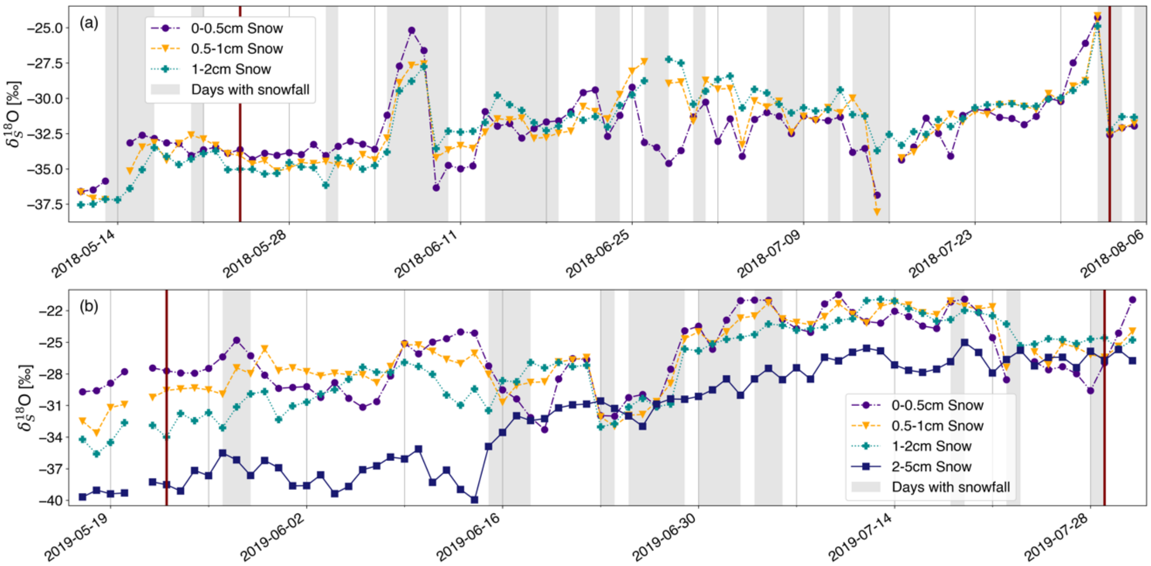

Fig. S1 The surface and sub-surface snow isotope variability

Timeseries of snow isotopic composition in different depths are presented for the 2018 and 2019 field season in (a) and (b), respectively. Note that the values presented are calculated from the snow samples (available on PANGAEA) that were sampled from the surface to the sampling depth under the assumption of homogenous mixing of the different layers.

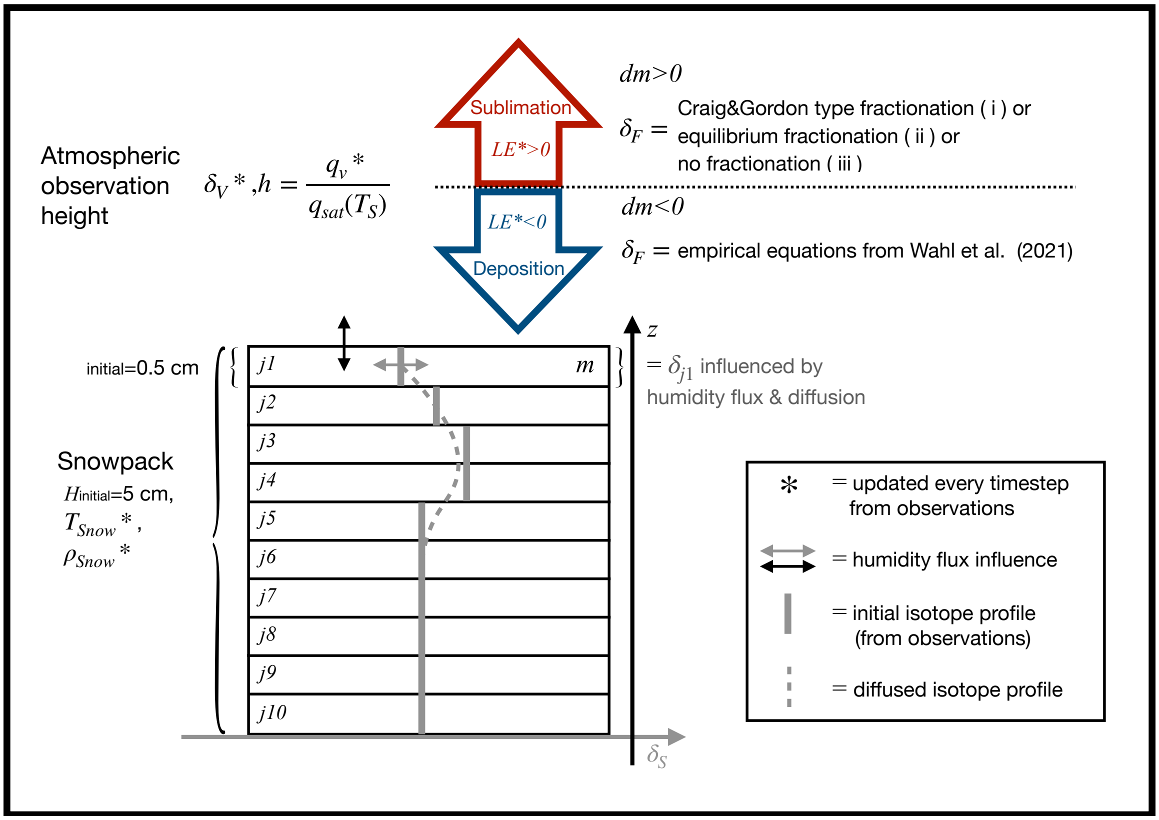

Fig. S2 Scheme of the snow surface model

The snowpack model domain is shown where the surface layer (j1) is influenced by the humidity flux (*LE*) and diffusion between snow layers (j). Model input parameters that are updated from observations are marked with *, while the other parameters are calculated from these observations. The snowpack isotope profile is initialized at the time of a snow sampling event and evolves driven by the humidity flux and diffusion until the time of the next sampling event the day after. The modeled change (**Δ**δ*) in the top snow layer's isotopic composition is then compared to the observed change.

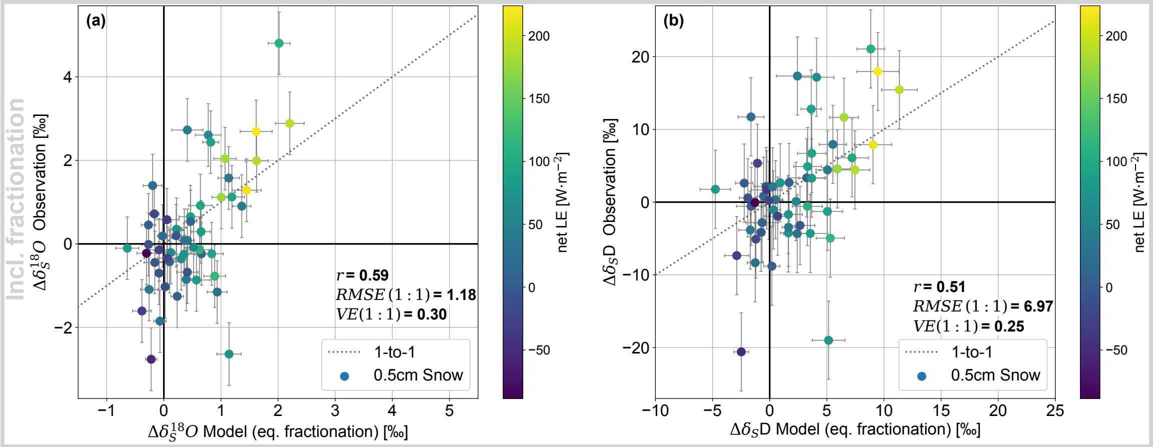

Fig. S3 Results of model experiment (ii) with equilibrium (eq.) fractionation Same as Figure 3 in the main text but assuming equilibrium fractionation during sublimation. On the x-axis the modeled day-to-day variability is shown for $\delta^{18}O$ in (a) and δD in (b). The observed $\boldsymbol{\Delta}\delta^*$ values are shown on the y-axis. All model periods (N=53) are color coded according to the cumulative *LE* observed between the respective two sampling events, bracketing the model period. The error bars for the model are computed as SD of the Monte Carlo runs and the uncertainty on the observations is calculated as described in Text 4.

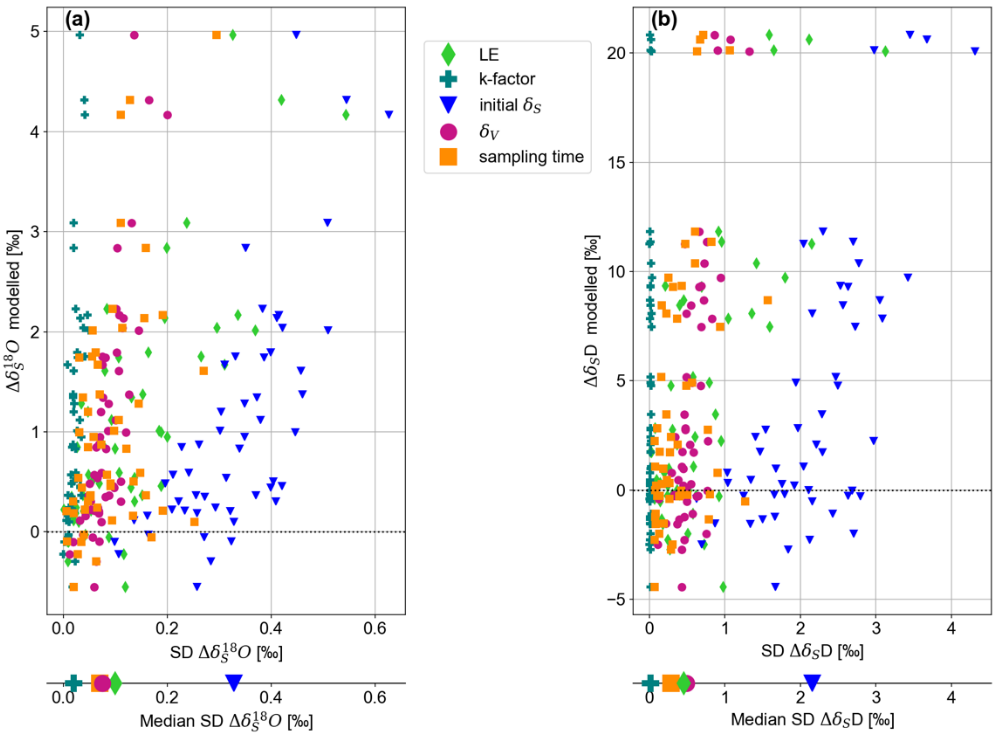

Fig. S4 Sensitivity analysis for different model input parameters
The model sensitivity for $\mathbf{\Delta}\delta^{18}$O in (a) and $\mathbf{\Delta}\delta$D in (b). The x-axis shows the SD of the distribution of $\mathbf{\Delta}\delta^{*}$ resulting when perturbing the input parameters of the model as shown in Table S2. The y-axis shows the modeled change in surface isotopic composition using the default input parameters. The median of all SD per disturbed parameter are shown on the lower axes and given in Table S2.

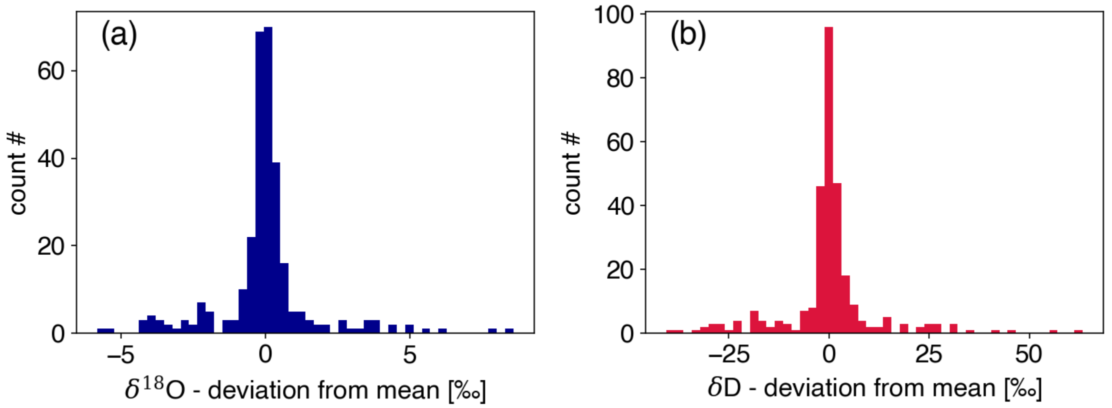

Fig. S5 Snow surface isotopic composition spatial variability
The deviations of the individual snow samples from the sample event average are plotted as histograms for $\delta^{18}O$ in (a) and δD in (b). From these distributions' standard deviations the uncertainty (SD) of the consolidated snow samples are inferred and yield a SD of 0.53‰ for $\delta^{18}O$ and 3.8‰ for δD as described in Text 4 of this Supplementary Material.

Table S1. The surface snow and vapor isotopic composition of the two observation periods 2018 and 2019 at EastGRIP. For the seasonal net change only days without snowfall and snowdrift were taken into account. The same days are used for the observed and modeled net change calculations.

		2018 (10 weeks)		2019 (9 weeks)	
		$\delta^{18}O$	δD	$\delta^{18}O$	δD
δ_s	Snow surface (0.5cm) mean	-32.25 ‰	-245.7 ‰	-26.29 ‰	-199.8 ‰
	Snow surface (0.5cm) standard deviation	3.32 ‰	23.5 ‰	2.43 ‰	19.6 ‰
	Snow surface (0.5cm) seasonal net change observed	9.84 ‰	59.7 ‰	4.33 ‰	30.4 ‰
	Snow surface (0.5cm) seasonal net change modeled excl. fractionation (experiment iii)	-3.85 ‰	-48.8 ‰	-1.56 ‰	-35.7 ‰
δ_v	Vapor (2m) mean	-47.39 ‰	-341.3 ‰	-41.52 ‰	-306.4 ‰

Table S2. Experiment results and sensitivity analysis for snow surface model

The model performance for the different experiments and model response to perturbed input parameters (± 1/ ± 2 uncertainty (SD)) is given in RMSE and VE both computed for the 1:1 line. Correlation coefficients *r* are given for the different experiments. The perturbation that yielded the highest VE value is shaded in green.

		δ^{18}O			δD		
**experiment**	**Model setting**	**RMSE [‰]**	**VE**	**r**	**RMSE [‰]**	**VE**	**r**
**(i)**	**no diffusion**	1.21	0.26	0.78	6.41	0.36	0.74
**(iii)**	**No Frac.**	1.33	0.11	0.44	8.24	-0.05	0.35
**(ii)**	**Eq. Frac.**	1.18	0.30	0.59	6.97	0.25	0.51
**(i)**	**CG**	1.13	0.36	0.82	5.52	0.53	0.78
**Sensitivity analysis**	**perturbation**	**RMSE [‰]**	**VE**	**median SD [‰]**	**RMSE [‰]**	**VE**	**median SD [‰]**
**δ_v 2m** **SD** for: δ^{18}O=0.23‰ δD=1.4‰	**+ 2SD**	1.22	0.25	0.08	5.87	0.47	0.49
	**+ SD**	1.18	0.31		5.68	0.50	
	**- SD**	1.09	0.40		5.38	0.55	
	**- 2SD**	1.05	0.44		5.28	0.57	
**δ_S initial** **SD** for: δ^{18}O=0.53‰ δD=3.8‰	**+ 2SD**	0.88	0.61	0.33	5.24	0.58	2.15
	**+ SD**	0.99	0.51		5.13	0.59	
	**- SD**	1.31	0.14		6.31	0.38	
	**- 2SD**	1.51	-0.14		7.38	0.16	
***k* factor**	**5e-3**	1.11	0.38	0.02	5.51	0.53	0.01
	**7e-3**	1.15	0.33		5.52	0.53	
LE	**+ 40%**	1.25	0.22	0.10	5.88	0.47	0.46
	**+ 20%**	1.19	0.29		5.67	0.50	
	**- 20%**	1.08	0.41		5.41	0.55	
	**- 40%**	1.04	0.45		5.41	0.55	
**Sampling time**	**+ 2h**	1.03	0.46	0.07	5.31	0.56	0.36
	**+ 1.5h**	1.06	0.44		5.36	0.56	
	**+ 1h**	1.08	0.41		5.41	0.55	
	**+ 0.5h**	1.11	0.38		5.47	0.54	
	**- 0.5h**	1.15	0.34		5.55	0.52	
	**- 1h**	1.17	0.31		5.59	0.52	
	**- 1.5h**	1.18	0.30		5.62	0.51	
	**- 2h**	1.19	0.28		5.64	0.51	
